# Cytokine Detection and Modulation in Acute Graft *vs*. Host Disease in Mice

**DOI:** 10.1155/S0962935194000062

**Published:** 1994

**Authors:** A. C. Knulst, G. J. M. Tibbe, C. Bril-Bazuin, E. G. Breedland, A. van Oudenaren, R. Benner, H. F. J. Savelkoul

**Affiliations:** Department of Immunology Erasmus University Rotterdam The Netherlands

## Abstract

A murine model for acute lethal graft *vs*. host disease (GVHD) was used to study the role that a number of cytokines play in the development of lethal GVHD. In this study we focused on the role of IL-1, IL-2, IL-4, IL-6, IFN-*γ* and TNF-*α*. Lethally irradiated (C57BL × CBA)F1 mice were reconstituted either with 10^7^ allogeneic BALB/c spleen cells or with a similar number of syngeneic cells, as a control. A significant rise in serum levels of IL-6, TNF-*α* and IFN-*γ* levels was found in allogeneically reconstituted mice. This is in contrast to the syngeneic control group in which no rise was seen. Serum IL-2 and IL-4 levels were below the detection limit. In the supernatant of Con A stimulated spleen cells from allogeneically reconstituted mice IL-6, IFN-*γ* and TNF-*α* concentrations were increased. The expression of mRNA for cytokines as detected by reverse transcription PCR was studied in spleen cells. In the allogeneic reconstituted mice the mRNA expression of IL-1*α*, IL-2, IL-6, IFN-*γ* and TNF-*α* displayed faster kinetics compared with that in syngeneic reconstituted mice. The effect of treatment with recombinant cytokines, antibodies to cytokines and to cytokine receptors on the development of GVHD was investigated. Administration of recombinant IL-2 to allogeneically reconstituted mice strongly increased the morbidity and mortality whereas injection of IL-1*α* and TNF-*α* did not influence survival. Administration of antibodies against IL-2 or the IL-2 receptor decreased the morbidity and mortality. Anti-IL-6, anti-IFN-*γ*, and anti-TNF-*α* mAB, on the other hand, did not affect the morbidity and mortality of GVHD. The results of this study suggest successive waves of cytokine-secreting cell populations consistent with the induction of an inflammatory response in the development of acute GVH disease.


Mediators of Inflammation 3, 33–40 (1994)

### Cytokine detection and modulation in acute graft *vs.* host disease in mice

A. C. Knulst, G. J. M. Tibbe, C. Bril-Bazuin, E. G. Breedland, A. van Oudenaren, R. Benner and H. F. J. Savelkoul[CA]

Department of Immunology, Erasmus University, Rotterdam, The Netherlands

[CA] Corresponding Author

**A MURINE model for acute lethal graft *vs.* host disease (GVHD) was used to study the role that a number of cytokines play in the development of lethal GVHD. In this study we focused on the role of IL-1, IL-2, IL-4, IL-6, IFN-γ and TNF-α. Lethally irradiated (C57BL × CBA)F1 mice were reconstituted either with $10^7$ allogeneic BALB/c spleen cells or with a similar number of syngeneic cells, as a control. A significant rise in serum levels of IL-6, TNF-α and IFN-γ levels was found in allogeneically reconstituted mice. This is in contrast to the syngeneic control group in which no rise was seen. Serum IL-2 and IL-4 levels were below the detection limit. In the supernatant of Con A stimulated spleen cells from allogeneically reconstituted mice IL-6, IFN-γ and TNF-α concentrations were increased. The expression of mRNA for cytokines as detected by reverse transcription PCR was studied in spleen cells. In the allogeneic reconstituted mice the mRNA expression of IL-1α, IL-2, IL-6, IFN-γ and TNF-α displayed faster kinetics compared with that in syngeneic reconstituted mice. The effect of treatment with recombinant cytokines, antibodies to cytokines and to cytokine receptors on the development of GVHD was investigated. Administration of recombinant IL-2 to allogeneically reconstituted mice strongly increased the morbidity and mortality whereas injection of IL-1α and TNF-α did not influence survival. Administration of antibodies against IL-2 or the IL-2 receptor decreased the morbidity and mortality. Anti-IL-6, anti-IFN-γ and anti-TNF-α mAB, on the other hand, did not affect the morbidity and mortality of GVHD. The results of this study suggest successive waves of cytokine-secreting cell populations consistent with the induction of an inflammatory response in the development of acute GVH disease.**

**Key words:** Cytokine detection, Cytokine modulation, Cytokine RT–PCR, Graft *vs.* host disease

### Introduction

Graft *vs.* host disease (GVHD) is the result of activation of transplanted donor T cells by alloantigens on host tissues.[1] The recognition of these antigens by T lymphocytes initiates a cascade of events which leads to the production of cytokines and the expression of their receptors. One of the first cytokines produced by activated T cells is IL-2. This cytokine enhances the expression of the IL-2 receptor and induces proliferation and activation of T cells.[2] Activated T lymphocytes differentiate into cells that produce a variety of cytokines.[3] This production of cytokines leads to recruitment and activation of other cell types such as B lymphocytes, macrophages and natural killer cells.[1,2] These cell types are involved in the process that leads to clinically overt GVHD and tissue destruction.

Recently, it was suggested that in murine models of acute and chronic GVHD, the development of acute and chronic GVHD associated pathological changes is related to differential cytokine production by activated T cells.[3] The subset of T cells activated and their cascades of sequential activation might be reflected in the set of cytokines that can be demonstrated during GVHD. For example, while T cell cytokines such as IL-4 and IL-10 play important roles in chronic GVHD, no clear role of these cytokines in acute models has been demonstrated yet.

The number of cytokines such as IL-1, IL-2, IFN-γ, TNF-α that are reported to play a role in experimental as well as clinical GVHD steadily increases.[4-8] The data from the literature, however, are not unequivocal. Moreover, many studies deal with only one particular cytokine. To improve current diagnostic and therapeutic approaches insight into the sequential involvement of the cells that play a role in GVHD, the cytokines they produce, their interactions and their regulation, is necessary.

The authors' previous investigations into the cellular aspects of murine GVHD is followed by this study of the role of cytokines, using a model of acute GVHD in lethally irradiated recipients in which $CD4^+$ T cells play a predominant role.[9,10]

© 1994 Rapid Communications of Oxford Ltd

For this study, serum samples and supernatants of spleen cell cultures were analysed for the presence of various cytokines, and spleen cell suspensions for the expression of mRNA for cytokines. The role of these cytokines in morbidity and mortality of GVHD was further assessed by treating allogeneically reconstituted mice with recombinant cytokines and monoclonal antibodies to cytokines and cytokine receptors.

### Materials and Methods

*Mice:* (C57BL/Ka × CBA/Rij)F1 (H-$2^{b/q}$) and BALB/c (H-$2^d$) mice were bred at the Department of Immunology of the Erasmus University. Recipient mice were age matched and 12–18 weeks old when entered in the experiments. During the experiments, mice were kept two per cage in light-cycled rooms and had access to acidified water and pelleted food *ad libitum*.

*Induction of GVHD:* GVH reactions were induced in lethally irradiated (10 Gy) mice by i.v. injection of $10^7$ allogeneic spleen cells within 24 h after irradiation. Irradiation was performed in a caesium-137 source (Gammacell 40, Atomic Energy of Canada, Ottawa, Canada) with a dose rate of 1.15 Gy/min. Mice were examined daily for the development of signs of GVHD, such as ruffled fur, hunched posture, decreased physical activity, wasting, skin lesions and diarrhoea, and for mortality. The body weight was determined two or three times per week during the first 4 weeks after GVHD induction. Thereafter the frequency was decreased depending on the health status of the mice. Mice judged moribund were killed. Irradiated control mice reconstituted with syngeneic cells survived >250 days without signs of disease. Radiation controls died between days 10 and 22.

*Collection of serum and tissue culture:* Mice were sacrificed using carbon dioxide. Blood collected by cardiac puncture was allowed to clot overnight at 4°C and centrifuged for 5 min in an Eppendorf centrifuge (International Equipment Company, Needham, MA). Serum samples were divided into aliquots and stored at −70°C. Spleen cell suspensions were prepared in BSS. Nucleated cell concentrations were determined with a Coulter Counter model ZB1. Viability of the cell suspensions as determined by the trypan blue exclusion method was >90%. For the determination of cytokines in spleen cell supernatants, spleen cells were cultured in RPMI 1640 tissue culture medium, supplemented with 10% FCS, glutamine and antibiotics according to Cleveland *et al.*[11] A concentration of $5 \times 10^5$ cells/well was seeded in five-fold in 96-well flat-bottom microtitre plates (Falcon, Becton Dickinson, Lincoln Park, NJ) and cultured for 24 h at 37°C and 5% $CO_2$ in the presence of Con A (1 μg/ml). The samples were centrifuged and the supernatants were collected and stored in aliquots at −70°C. For each cytokine all samples were assayed simultaneously.

*Determination of cytokines:* IL-2, IL-6 and TNF-α were determined in a bioassay using CTLL-2, B9 and WEHI164 (clone 13) indicator cells respectively, as described previously.[12–14] The MTT assay was used to quantitate the proliferative and cytotoxic activity.[15] The procedure was described previously with minor modifications.[16] Briefly, 30 μl MTT solution (5 mg/ml 3-(4,5-dimethylthiazol-2-yl)-2,5-diphenyl tetrazolium bromide (Sigma, St Louis, MO, in PBS) was added to each well. Plates were incubated for 3.5 h at 37°C with 5% $CO_2$. The supernatant was discarded and 100 μl DMSO (Merck, Darmstadt, Germany) was added to each well. The plates were monitored on a Titertek Multiskan MCC 96-well ELISA plate reader (Flow Laboratories, Ayrshire, Scotland) at a wavelength of 510 nm. The experimental values were corrected for background values. IFN-γ was determined in ELISA as described.[17]

*Detection of mRNA for cytokines:* RNA was isolated from spleen cell preparations using guanidinium thiocyanate extraction in combination with gradient centrifugation on 5.7 M caesium chloride for 18 h in an ultracentrifuge (Sorvall, Du Pont, Newtown, CT) as described.[15] After ethanol precipitation 1 μg RNA was used in a reverse transcriptase reaction. For amplification 35 cycles (1 min at 94°C for denaturation, 2 min at 55°C for annealing and 3 min at 72°C for primer extension) were performed, using a DNA thermal cycler (Perkin–Elmer, Gouda, The Netherlands). For all samples sense and anti-sense primer sets specific for IL-1α, IL-2, IL-6, IFN-γ, TNF-α were used as described.[18] A primer set specific for the housekeeping gene hypoxanthine phosphatidyl ribosyltransferase (HPRT) was used as an internal control.

*Recombinant cytokines:* Recombinant cytokines were administered *in vivo* by s.c. injection in the inguinal region, in a volume of 0.25–0.5 ml. Recombinant (r) IL-2 was kindly provided by Dr G. Malainer (Sandoz Forschungsinstitut, Wien, Austria). IL-2 was shown to enhance murine CTLL-2 proliferation based bio-assay and its activity could be neutralized effectively by addition of anti-IL-2 antibodies (S4B6.1).[19] Human IL-1α was a gift from Hoffman–La Roche, Mijdrecht, The Netherlands. Recombinant murine (rm) IFN-γ was a kind gift of Dr R. L. Coffman (DNAX Research Institute, Palo Alto, CA). The rmIL-6 was purchased from British Biotechnology, Abingdon, UK. Human rTNF-α was a gift from BASF/Knoll, Ludwigshafen, Germany.

*LPS and antibodies:* LPS-B (*Salmonella typhosa* 0901) was purchased from Difco Laboratories, Detroit, MI. Mice received a dose of 100 μg i.p. dissolved in BSS.[20]

For *in vivo* treatment, anti-IL-2 (S4B6.1),[19] anti-IL-6 (MP 20F3),[21] anti-IFN-γ (XMG 1.2)[17] and anti-TNF-α (XT22),[21] producing hybridomas were used to purify antibodies from. An anti-IL-2R (PC61) producing hybridoma was obtained from the American Type Culture Collection. The mAb were purified from ascites by protein G (Pierce Europe, Oud-Beijerland, The Netherlands) affinity chromatography and subsequently administered by i.p. injection in doses up to 1 mg. Rat IgG1 in the serum of anti-cytokine antibody treated mice was determined by a specific ELISA.[22]

*Statistical analysis:* Differences between groups were analysed using the Student's *t*-test or the Wilcoxon–Mann–Whitney statistic. Values of $p < 0.05$ were considered significant.

### Results

*Serum cytokine levels:* Groups of (C57BL × CBA)F1 mice were lethally irradiated and reconstituted with either $10^7$ allogeneic BALB/c or $10^7$ syngeneic spleen cells as controls. Signs of disease were detectable in the allogeneically reconstituted mice from day 6 onwards, whereas mortality occurred from day 8 onwards. The mean survival time of these mice was 12.5 ± 4.9 days. The syngeneically reconstituted mice survived > 250 days without any symptom of disease.

To demonstrate that T cells were involved in the production of IFN-γ, mice ($n = 5$) were treated with a single i.p. dose of 100 μg anti-Thy-1 at 24 h after reconstitution with allogeneic or syngeneic spleen cells.[9] Serum IFN-γ levels at day 7 were 22 ± 5 μg/ml in the allogeneic group. The serum IFN-γ levels in anti-Thy-1 treated allogeneically reconstituted mice were 6 ± 3 μg/ml and comparable with those of the syngeneic group (4 ± 2 μg/ml).1 These data show that the observed serum IFN-γ was produced by T cells.

In order to examine the cytokine production, five mice from both groups were sacrificed at 1, 2, 3, 4, 5, 6, 7, 8 and 10 days after reconstitution. Serum samples from these mice were analysed for the presence of IL-2, IL-4, IL-6, IFN-γ and TNF–α. Figure 1A illustrates the IL-6 levels in serum, which increased at day 4 after allogeneic reconstitution. From day 5 onward these IL-6 levels were significantly increased ($p < 0.05$) compared with the syngeneically reconstituted mice in which no rise in IL-6 levels was seen. Serum IFN-γ levels in the allogeneically reconstituted mice increased at day 4 with a peak on day 5 and 6 (Fig. 1B). On the peak days these levels were significantly higher ($p <$

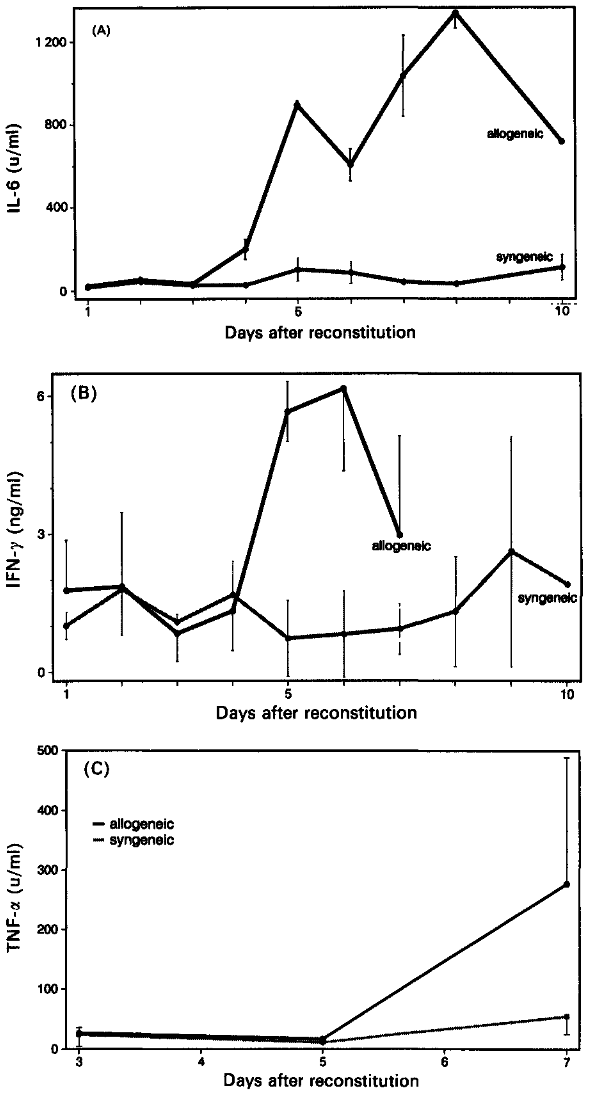

FIG. 1. Serum IL-6, IFN-γ and TNF-α levels in acute GVHD. Lethally irradiated (C57BL × CBA)F1 mice were reconstituted either with $10^7$ allogeneic BALB/c spleen cells or $10^7$ syngeneic spleen cells. From each of these two groups the serum levels of IL-6 (A) and IFN-γ (B) was examined from day 1 until day 10 after reconstitution. TNF-α serum levels (C) were determined on days 3, 5 and 7. Each point represents the arithmetic mean ± 1 S.E.M. ($n = 5$).

0.05) compared with the syngeneically reconstituted group in which no rise of IFN-γ was seen. In the allogeneic group, TNF-α levels were significantly higher on day 7 only when compared with the syngeneic control (Fig. 1C). IL-2 and IL-4 levels were below the detection limit: < 0.1 U/ml (data not shown).

*Cytokines in spleen cell supernatants:* Since cytokine levels in the serum are considered an indirect reflection of the cytokine production in the spleen, we were interested in the cytokine production by the spleen cells themselves at various times after induction of GVHD. Therefore, lethally irradiated (C57BL × CBA)F1 mice were reconstituted with either $10^7$ BALB/c or syngeneic spleen cells. On days 3, 5 and 7 after reconstitution spleen cells from three mice of each group were cultured in the presence of Con

A. After 24 h culture, levels of IL-2, IL-4, IL-6, IFN-$\gamma$ and TNF-$\alpha$ were determined in the supernatants ($n = 3$). Figure 2 (A–C) shows that IL-6, IFN-$\gamma$ and TNF-$\alpha$ levels in the allogeneically reconstituted group were significantly increased ($p < 0.05$) compared with syngeneically reconstituted mice. While the production of IFN-$\gamma$ and TNF-$\alpha$ were peaking at day 5 after induction of GVHD, IL-6 production decayed from an initial high production at day 3. Preliminary data substantiate these increased productions by finding increased frequencies of IL-6, IFN-$\gamma$ and TNF-$\alpha$ secreting cells using newly developed ELISA plaque assays (data not shown).

*Detection of cytokine mRNA:* From both groups of mice spleen cell suspensions were analysed for the presence of cytokine mRNA on days 1, 2, 4, 5 and 7 after reconstitution indicating the actual production of these cytokines *in vivo*. Figure 3 shows a

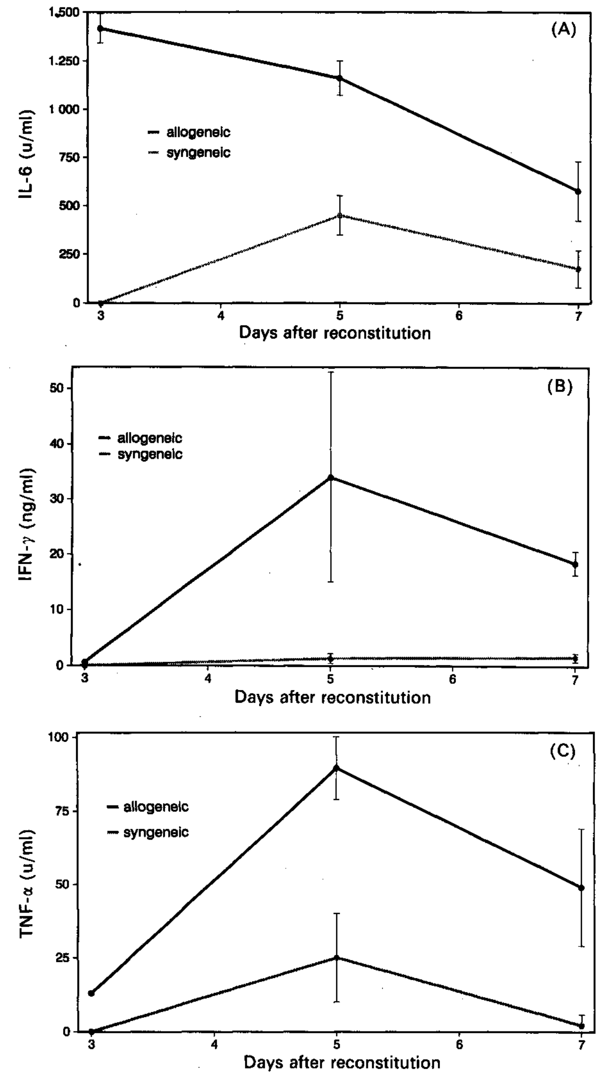

FIG. 2. IL-6 (A), IFN-$\gamma$ (B) and TNF-$\alpha$ (C) levels in the supernatant of 24 h spleen cell cultures. From allogeneically and syngeneically reconstituted mice spleen cells were cultured for 24 h in the presence of Con A. Each point represents the arithmetic mean of the cytokine level $\pm$ 1 S.E.M. ($n = 3$).

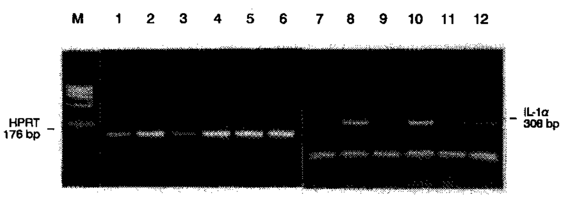

FIG. 3. Expression of IL-1 mRNA in lethally irradiated (C57BL $\times$ CBA)F1 mice reconstituted with either $10^7$ allogeneic BALB/c spleen cells or syngeneic F1 spleen cells. Total splenic RNA was extracted on days 1, 2, 4, 5 and 7, cDNA was prepared and reverse type PCR analysis was performed using an HPRT primer set (lanes 1–6) or an IL-1$\alpha$ primer set (lanes 7–12). Lane M: base pair marker ($\phi \times 174$ RF DNA Hae III digest), lanes 1 and 7, 3 and 9, 5 and 11 represent syngeneically reconstituted mice analysed on days 4, 5 and 7, respectively. Lanes 2 and 8, 4 and 10, 6 and 12 represent allogeneically reconstituted mice analysed on days 4, 5 and 7, respectively.

typical result of the RT–PCR analysis of IL-1$\alpha$ mRNA expression in both syngeneically and allogeneically reconstituted mice. Allogeneically reconstituted mice displayed an early and abundant expression of IL-1$\alpha$ starting from day 1 after reconstitution. Expression of IL-1$\alpha$ mRNA could also be detected in syngeneically reconstituted animals only by day 7. The results of this analysis are summarized in Table 1. As shown in Table 1, in the allogeneically reconstituted mice mRNA levels of IL-1$\alpha$, IL-6 and TNF-$\alpha$ were detectable from day 1, while mRNA levels of IL-2 and IFN-$\gamma$ were detectable at days 4 and 5, respectively, by using this RT–PCR analysis. Until day 7 these cytokines were not detectable in the syngeneically reconstituted group. On day 7 the mRNA levels were similar in both groups.

*Effect of administration of recombinant cytokines:* Based upon the expression in the early development of GVHD of IL-1$\alpha$, IL-2 and TNF-$\alpha$ these cytokines were chosen to modulate the developing signs of GVHD. We investigated the effect of administration of rIL-2 to (C57BL $\times$ CBA)F1 mice that were lethally irradiated and reconstituted with $10^7$ BALB/c spleen cells. The morbidity in both groups was comparable. IL-2 was injected twice daily at a dose of 50 000 IU, either from days 0 to 3 or alternatively, from days 6 to 9 after reconstitution based on the studies of Sykes *et al.*[23] The results are shown in Fig. 4A. IL-2 administration from days 6 to 9 resulted in increased mortality compared with the non-treated control group. However, since the GVHD in this experiment was more chronic than in other experiments using the same strain combinations this is not considered a relevant effect. IL-2 injection from days 6 to 9 on the other hand resulted in a significantly increased mortality. Treatment with a lower dose of 5 000 IU twice daily did not influence the morbidity or mortality in this model of GVHD (data not shown).

The effect of rhIL-1 was studied after a single dose of 400 ng of IL-1$\alpha$ on day $-1$, 3 or 6. It appeared that IL-1$\alpha$ treatment did not influence

**Table 1.** mRNA expression for IL-1α, IL-2, IL-6, IFN-γ and TNF-α during GVHD

| | Days after reconstitution | | | | | | | | | |
|---|---|---|---|---|---|---|---|---|---|---|
| | 1 | | 2 | | 4 | | 5 | | 7 | |
| Cytokine | allo | syn | allo | syn | allo | syn | allo | syn | allo | syn |
| IL-1α | + | − | + | − | + | − | + | − | ± | ± |
| IL-2 | − | − | − | − | + | − | + | − | + | + |
| IL-6 | ± | − | ± | − | + | − | + | − | − | ± |
| IFN-γ | − | − | − | − | − | − | + | − | ± | ± |
| TNF-α | ± | − | + | − | + | − | + | − | ± | + |

(C57BL × CBA)F1 mice were lethally irradiated reconstituted with $10^7$ allogeneic (allo) BALB/c or syngeneic (syn) spleen cells. At days 1, 2, 4, 5 and 7 after reconstitution, pooled spleen cells of both groups were analysed for the expression of mRNA for IL-1α, IL-2, IL-6, IFN-γ and TNF-α ($n = 5$).
+ = strongly visible signal, ± = weakly visible signal, − = no visible pcr product.

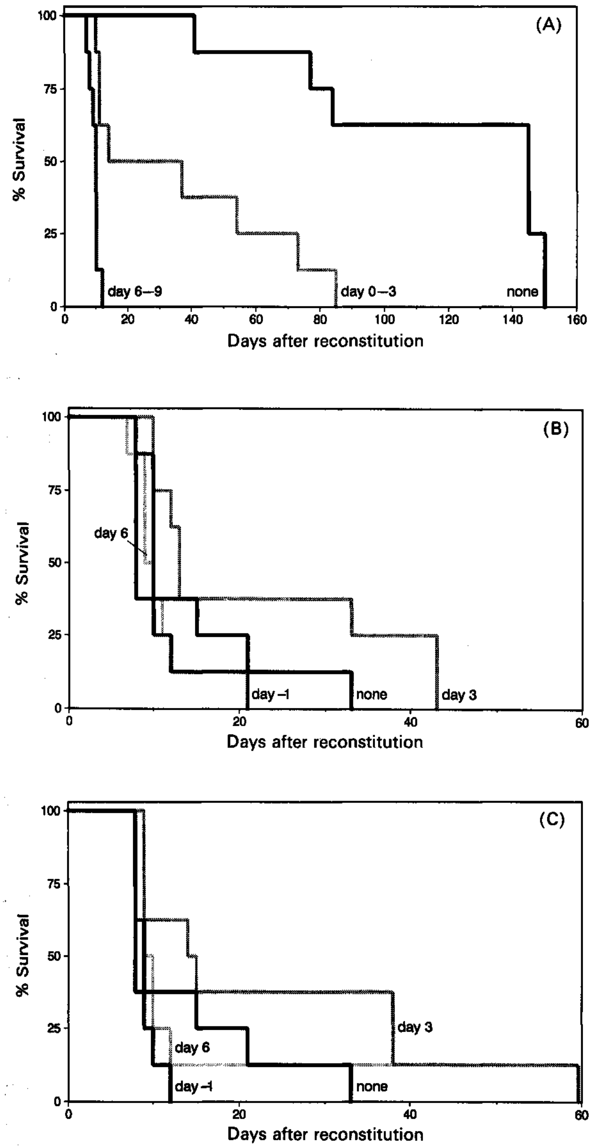

FIG. 4. (A) Effect of administration of rIL-2 *in vivo* on GVHD. Lethally irradiated (C57BL × CBA)F1 mice were reconstituted with $10^7$ BALB/c spleen cells and subsequently treated twice daily with 50000 IU rIL-2 s.c. at days 0 to 3 or 6 to 9 after reconstitution ($n = 8$). (B) Effect of rIL-1α. GVHD was induced as indicated. A dose of 400 ng of rIL-1 was given either on days −1, 3 or 6 after reconstitution ($n = 8$). (C) Effect of rTNF-α. GVHD was induced as indicated. A dose of 2 μg of rTNF-α was given on either day −1, 3 or 6 after reconstitution ($n = 8$).

the survival (Fig. 4B). The effect of a single dose of 2 μg of rhTNF-α was studied after injection on either day −1, 3 or 6. TNF-α administration had no effect on the survival (Fig. 3).

*Effect of injection of LPS: In vivo* injection of LPS is known to induce IL-1, IL-6 and TNF-α production in a time dependent fashion. LPS might therefore modulate the course of GVHD.[6] We studied whether injection of LPS at various days after reconstitution influenced the mortality of GVHD in the BALB/c–(C57BL × CBA)F1 strain combination in three independent experiments. In a representative experiment, groups of mice received 100 μg LPS either on day −1, 0, 1, 3 or 6 after allogeneic reconstitution. It appeared that the effect of LPS administration was dependent on the time of injection (Fig. 5). LPS did not enhance GVHD and mortality when injected on day 1, 3 or 6 after reconstitution. The effect appeared stronger when the LPS was administered at a later time point after reconstitution. In contrast, injection of LPS on day −1 appeared to inhibit GVHD. In all experiments, a rather acute GVHD was induced in the non-treated allogeneically reconstituted mice.

*Effect of anti-cytokine and anti-cytokine receptor mAB:* (C57BL × CBA)F1 mice were lethally irradiated,

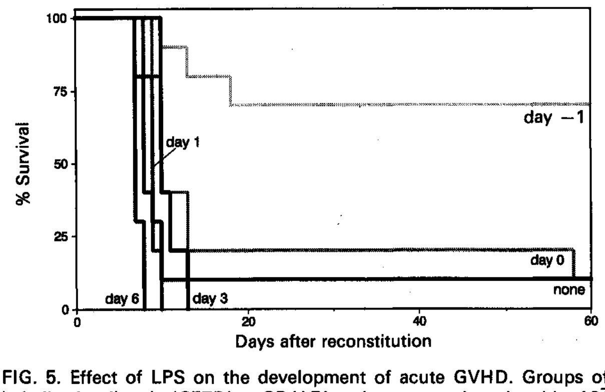

FIG. 5. Effect of LPS on the development of acute GVHD. Groups of lethally irradiated (C57BL × CBA)F1 mice reconstituted with $10^7$ BALB/c spleen cells were i.p. injected with 100 μg of LPS on either day −1, 0, 1, 3 or 6 after reconstitution ($n = 10$).

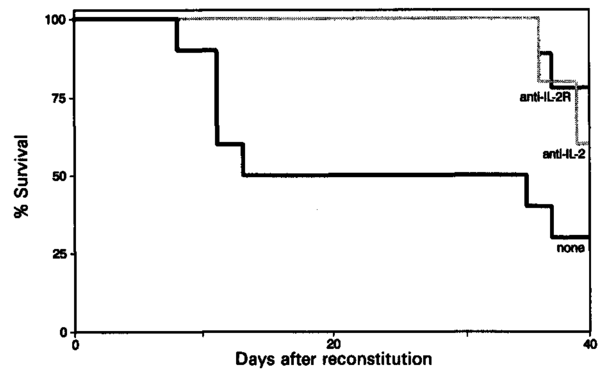

FIG. 6. Effect of anti-IL-2 and anti-IL-2R mAb treatment. Lethally irradiated (C57BL x CBA)F1 mice reconstituted with $10^7$ BALB/c spleen cells were injected with 0.1 ml anti-IL-2 ascitic fluid or 1 mg anti-IL-2R mAb 1 day after reconstitution ($n = 10$).

reconstituted with $10^7$ BALB/c spleen cells and injected the day after reconstitution with rat antibodies against several cytokines in doses up to 1 mg that have been widely shown to be sufficient to neutralize the respective cytokine activities in a variety of systems. Upon treatment with these mAb detectable levels (>1 μg/ml) of rat IgG occurred for a period up to 14 days (data not shown).

Combinations of several anti-cytokine antibodies specific for TNF-α, IFN-γ and IL-6 did not result in a significant increase of the survival. Anti-IL-2 and anti-IL-2R mAb, however, did affect the development of GVHD. These mAb were injected the day after reconstitution. As shown in Fig. 6, both mAb were able to enhance the survival significantly ($p < 0.05$). However, mortality did occur, starting on day 38. This indicates that the inhibitory effect of anti-IL-2 and anti-IL-2R mAb was only temporary at the dose tested.

### Discussion

The main finding in this study is that in developing acute GVHD the production of IL-6, IFN-γ and TNF-α is significantly increased although IL-2 is the crucial cytokine which acts as a target for intervention studies. Most studies on the role of cytokines in GVHD deal with only one particular cytokine. We studied the role of several cytokines that are potentially involved in GVHD, by cytokine mRNA expression, analysis of cytokines in serum and culture supernatant and by *in vivo* administration of recombinant cytokines or anti-cytokine and anti-cytokine receptor mAb. These data substantiate the important role that cytokines play in GVHD.[24] In GVHD, a role for IL-1, IL-2, IFN-γ and TNF-α ('cytokine storm') has been postulated.[4–8] Since IL-1 is produced in increasing amounts as clinical GVHD progresses, blocking of IL-1 activity led to strongly enhanced survival from GVHD in mice.[8,25] Blocking of IL-1 might interfere with early T-cell activation.[2] In the murine model used, we have earlier demonstrated that $CD4^+$ T cells play a dominant role in the induction of GVHD.[9] Here we show by anti-Thy-1 treatment that the production of some cytokines (IFN-γ) is dependent on T cells. In this study treatment with IL-1 did not result in enhanced symptoms of GVHD, which might be caused by the dose used. On the other hand, conflicting data exist in which treatment with 10 μg IL-1 resulted in enhanced survival in lethally irradiated and allogeneically reconstituted mice.[26] With the development of cytokine ELISA plaque assays increased frequencies of cytokine-secreting cells were observed in con A stimulated spleen cell cultures. These data together with the observed low, but relatively constant cytokine production rate per cell,[27] argue for a higher proportion of IL-6, IFN-γ and TNF-α secreting cells in the spleens of mice suffering from GVHD.

A surprising result was the sequential cytokine mRNA expression specific for the allogeneically reconstituted mice by RT–PCR analysis. The differential kinetics of the cytokine expression resulted in early expression of IL-1α, IL-6 and TNF-α (day 1) while IL-2 and IFN-γ were found only later (days 4 and 5). In the syngeneic group cytokine gene expression was not detected before day 7. The kinetics of the cytokine mRNA expression is reflected in the production found in Con A supernatants for IL-6, TNF-α and IFN-γ. The detection of these cytokines in the serum is delayed by several days. The peak of IFN-γ activity coincided with a strong increase in the number of $CD4^+$ T cells in the spleen[10] and also with DTH reactivity.[28] This might reflect Th1 activity, as an important event in the development of GVHD. The peak of IL-6 activity coincided with a strong increase in the number of $CD8^+$ T cells.[10] These observations might reflect the sequential involvement of several cell populations like activated monocytes/macrophages, $CD4^+$ T cells and possibly $CD8^+$ T cells and their cytokines in our model.[24] While the findings in this study suggest an important role for monokines in mediating acute GVHD, it must be remembered that GVHD is initiated by T cells.[3,10] Ford *et al.* have shown in a sponge matrix allograft model that the occurrence of IL-6 coincided with cytotoxic T cell development.[29] A number of studies suggests that also TNF-α plays an important role in the pathogenesis of GVHD.[7,30,31] elevated serum levels of TNF-α preceded clinical symptoms of GVHD. A possible explanation for the difference between the cytokine gene expression and production in the syngeneic and allogeneic group could be a higher frequency of cytokine-producing cells in the allogeneic group. This possibility is currently under investigation. So far, few studies have been described aiming at modulating GVHD by treatment with recombinant cytokines rather than anti-cytokine antibodies or receptor agonists *in vivo*.[26,32]

The impact of the balance between Th1 and Th2 cells for the induction and pathogenesis of GVHD is still unclear. Several groups found evidence for predominance of a Th2 response in a model for chronic GVHD.[33,34] In our model IL-2 and IL-4 were not detectable in the serum, possibly as a result of rapid consumption. However, the involvement of IL-2 is implicated by: (a) the finding of enhanced IL-2 mRNA expression after allogeneic reconstitution; (b) the observation that exogenous IL-2 enhanced the mortality; and (c) the observation that anti-IL-2 and anti-IL-2R mAb were able to decrease the morbidity and mortality of GVHD. Enhanced mortality of GVHD by exogenous IL-2 administration was also reported by others.[1,23,32] The fact that IL-2 administration at a later stage (days 6 to 9) enhanced the mortality more strongly than IL-2 administered during the first few days after reconstitution, suggests that the strongest effect of exogenous IL-2 is exerted on $CD8^+$ T cells. However, $CD4^+$ T cells are activated and give rise to large amounts of IL-2 only in the early phase of GVHD (starting from day 4). They might be less dependent on exogenous IL-2, since they can produce IL-2 themselves. This IL-2 leads to expansion of $CD8^+$ T cells, which is the dominant cell population in the development of GVHD. Sykes *et al.* found that IL-2 treatment given early after allogeneic reconstitution even delayed the development of GVHD.[23] This effect was most clearly found in chronic GVHD and was dependent on the dose of IL-2. Recently, these authors were able to demonstrate that early IL-2 administration had an inhibitory effect on donor T cells.[32] Apparently, in our model of acute GVHD such a beneficial effect of early IL-2 administration did not occur. The concept that activation of Th1 and Th2 subsets led to differential cytokine production and subsequently disease associated pathology may also underline the varied manifestations of GVHD.[3] The balance between Th1 cells (mediating inflammatory and cytotoxic T cell responses) and Th2 cells (involved in humoral immunity) will thus determine the acuteness or chronicity of the pathological changes in induced GVHD.

The administration of LPS, which is known to induce the sequential production of a number of cytokines, such as IL-1, IL-6 and TNF-α,[35] also influenced the morbidity and mortality of GVHD. Since LPS injection enhanced the development of GVHD and mortality the results substantiate the relevance of these cytokines detected in the serum of allogeneically reconstituted mice. This finding is consistent with the hypothesis of an inflammatory cytokine production as the common pathway of acute GVHD.[36] During GVHD macrophages become primed as the result of the allogeneic reaction and subsequent production of TNF-α and possibly IL-1 is triggered.[25] The earlier after reconstitution that the LPS was administered, the less the mortality was enhanced. Administration of LPS one day before allogeneic reconstitution even delayed the development of GVHD. We also studied the effect of exogenous IL-1 and TNF-α administration. However, it appeared that at the dose tested, neither IL-1 nor TNF-α influenced the survival. It is tempting to speculate that this is due to the presence of IL-1 receptor antagonist and soluble TNF-α receptors in the serum. These results, however, could be compromised by the rather acute GVHD in the control mice thereby obscuring some possible activities mediated by LPS. On the other hand, the dose of LPS used was sufficiently high to expect strong *in vivo* effects.[20]

More insight into the involvement of cytokines in GVHD can also be obtained by studying the effect of neutralizing mAb directed to cytokines or their receptors. We studied the effect of anti-IL-6, anti-IFN-γ and anti-TNF-α mAb. Even repeated or combined administration of these mAb in doses that are known to exert distinct *in vivo* effects[21,32,35,37] did not influence the morbidity or mortality of GVHD in our model. Thus far, most reports dealing with administration of a single mAb to a particular cytokine show that a beneficial effect depends on co-administration of immunosuppressive agents, such as cyclosporine A.[38] Also, in human bone marrow transplantation treatment with a monoclonal anti-TNF-α antibody in combination with cyclosporin was found to be ineffective in preventing GVHD in the majority of cases.[39] It has also been described that specific lesions, e.g. gut lesions, due to GVHD can improve upon anti-cytokine (anti-IFN-γ) treatment only, but decreased mortality has not been found.[4,33] It can be speculated that once a GVH reaction has been initiated, a variety of cytokines is released ('cytokine storm') which have overlapping activities based on the degeneracy in the cytokine network. Blocking of the activity of only one particular cytokine will be ineffective in that case. Another possibility is that the local amount of cytokines produced in GVHD is so high that even higher systemic doses are required. Finally, it cannot be excluded that in GVHD cytokines other than those studied here, are involved. Recently, IL-7 was reported to be involved in tumour rejection[40] whereas IL-8 levels were found to be elevated after liver transplantation.[41]

The observation that anti-IL-2 mAb so far is the only anti-cytokine mAb that is able to enhance the survival might be explained by the critical role of IL-2 in T cell activation.[2] Similarly, anti-IL-2R mAb were able to reduce the morbidity and mortality of GVHD. It is likely that blockade of the cytokine cascade is most easy during the induction phase. These data point to a critical role for IL-2 as a possible therapeutic target in the prevention of GVH. Preliminary data in humans show also a beneficial effect of anti-IL-2R treatment in GVHD.[42] The results of serum and culture supernatant analyses suggest that, besides IL-2, IL-6, IFN-$\gamma$ and TNF-$\alpha$ are involved as well. The observation that serum IL-6, IFN-$\gamma$ and TNF-$\alpha$ levels are elevated, before symptoms of GVHD become apparent, suggests that careful evaluation of cytokines in serum might be useful for the early detection of GVHD. It has to be stressed, however, that elevation of cytokine levels is not restricted to alloreactivity but occurs also during bacterial and viral infections and that determination of cytokines at present can be used only to support current diagnostic procedures.

### References

1. Ferrara JLM, Deeg HJ. Graft-versus-host disease. *N Engl J Med* 1991; **324**: 667.
2. Krensky AM, Weiss A, Crabtree G, Davis MM, Parham P. T lymphocyte-antigen interactions in transplant rejection. *N Engl J Med* 1990; **322**: 510.
3. Allen RD, Staley TA, Sidman CL. Differential cytokine expression in acute and chronic murine graft-versus-host disease. *Eur J Immunol* 1993; **23**: 333.
4. Mowat AM. Antibodies to IFN-$\gamma$ prevent immunologically mediated intestinal damage in murine graft-versus-host reaction. *Immunology* 1989; **68**: 18.
5. Malkovsky M, Brenner MK, Hunt R, *et al.* T cell depletion of allogeneic bone marrow prevents acceleration of graft-versus-host disease induced by exogenous interleukin-2. *Cell Immunol* 1986; **103**: 476.
6. Cohen J. Cytokines as mediators of graft-versus-host disease. *Bone Marrow Transplant* 1988; **3**: 193.
7. Holler E, Kolb HJ, Möller A, *et al.* Increased serum levels of tumor necrosis factor-$\alpha$ precede major complications of bone marrow transplantation. *Blood* 1990; **4**: 1011.
8. McCarthy PL, Abhyankar S, Neben S, *et al.* Inhibition of interleukin-1 by an interleukin-1 receptor antagonist prevents graft-versus-host disease. *Blood* 1991; **78**: 1915.
9. Knulst AC, Bril-Bazuin C, Benner R. Prevention of lethal graft-versus-host disease by a single low dose of anti-T cell monoclonal antibody to the allograft recipients. *Eur J Immunol* 1991; **21**: 103.
10. Knulst AC, Tibbe GJM, Bril-Bazuin C, Benner R. Improved survival from potentially lethal graft-vs.-host disease by donor pretreatment with a recipient-specific blood transfusion. II. Evidence for a principal role of CD4$^+$ T cell subset. *Eur J Immunol* 1993; **23**: 299.
11. Cleveland MG, Annable CR, Klimpel GR. *In vivo* and *in vitro* production of IFN-$\beta$ and IFN-$\gamma$ during graft-versus-host disease. *J Immunol* 1988; **141**: 3349.
12. Gillis S, Ferm MM, Ou W, Smith KA. T cell growth factor: parameters of production and a quantitative microassay for activity. *J Immunol* 1978; **120**: 2027.
13. Aarden LA, de Groot ER, Schaap OL, Lansdorp PM. Production of hybridoma growth factor by human monocytes. *Eur J Immunol* 1987; **17**: 1411.
14. Meager A, Leung H, Woolley J. Assays for tumour necrosis factor and related cytokines. *J Immunol Meth* 1989; **116**: 1.
15. Chirgwin JM, Przybyla AE, MacDonald RJ, Gasson JC. Isolation of biologically active ribonucleic acid from sources enriched in ribonuclease. *Biochemistry* 1979; **18**: 5294.
16. Mosmann T. Rapid colorimetric assay for cellular growth and survival: application to proliferation and cytotoxicity assays. *J Immunol Meth* 1983; **65**: 55.
17. Cherwinski HM, Schumacher JH, Brown KD, Mosmann TR. Two types of mouse helper T cell clone. III. Further differences in lymphokine synthesis between Th1 and Th2 clones revealed by RNA hybridization, functionally monospecific bioassays and monoclonal antibodies. *J Exp Med* 1987; **166**: 1229.
18. Murray LJ, Lee R, Martens C. *In vivo* cytokine gene expression in T cell subsets of the autoimmune MRL/Mp-*lpr/lpr* mouse. *Eur J Immunol* 1990; **20**: 163.
19. Mosmann TR, Cherwinski H, Bond MW, Giedlin MA, Coffman RL. Two types of murine helper T cell clone: I. Definition according to profiles of lymphokine activities and secreted proteins. *J Immunol* 1986; **136**: 2348.
20. Molendijk WJ, Ploemacher RE, Erkens-Versluis ME. Mediatory role of stem cell derived cells in LPS-induced splenic $CFU_2$ accumulation. *Exp Hematol* 1982; **10**: 499.
21. Starnes Jr HF, Pearce MK, Tewari A, Yim JH, Zou J-C, Abrams JS. Anti-IL-6 monoclonal antibodies protect against lethal *Escherichia coli* infection and lethal tumor necrosis factor-$\alpha$ challenge in mice. *J Immunol* 1990; **145**: 4185.
22. Savelkoul HFJ, Ommen R van, Vossen ACTM, Breedland EG, Coffman RL, Oudenaren A van. Modulation of systemic cytokine levels by implantation of alginate encapsulated cells. Submitted for publication.
23. Sykes M, Romick ML, Hoyles KA, Sachs DH. *In vivo* administration of interleukin-2 plus T cell-depleted syngeneic marrow prevents graft-versus-host disease and permits alloengraftment. *J Exp Med* 1990; **171**; 645.
24. Jadus MR, Wepsic HT. The role of cytokines in graft-versus-host reactions and disease. *Bone Marrow Transpl* 1992; **10**: 1.
25. Ferrara JLM, Abhyankar S, Gilliland DG. Cytokine storm of graft-versus-host disease: a critical effector role for interleukin-1. *Transpl Proc* 1993; **25**: 1216.
26. Oppenheim JJ, Neta R, Tiberghien P, Gress R, Kenny JJ, Longo DL. Interleukin-1 enhances survival of lethally irradiated mice treated with allogeneic bone marrow cells. *Blood* 1989; **74**: 2257.
27. Shirai A, Holmes K, Klinman D. Detection and quantification of cells secreting IL-6 under physiological conditions in BALB/c mice. *J Immunol* 1993; **150**: 793.
28. Benner R, Wolters EAJ, Bril H, Molendijk A, van Oudenaren A. Regulation of delayed-type hypersensitivity to host histocompatibility antigens during graft-versus-host reactions. *Immunol Rev* 1985; **88**: 25.
29. Ford HR, Hoffman RA, Tweardy DJ, Kispert P, Wang S, Simmons RL. Evidence that production of interleukin-6 within the rejecting allograft coincides with cytotoxic T lymphocyte development. *Transplantation* 1991; **51**: 656.
30. Piguet P-F, Grau GE, Allet B, Vassalli P. Tumor necrosis factor/cachectin is an effector of skin and gut lesions of the acute phase of graft-versus-host disease. *J Exp Med* 1987; **166**: 1280.
31. Symington FW, Pepe MS, Chen AB, Deliganis A. Serum tumor necrosis factor-$\alpha$ associated with acute graft-versus-host disease in humans. *Transplantation* 1990; **50**: 518.
32. Abraham VS, Sachs DH, Sykes M. Mechanism of protection from graft-versus-host disease mortality by IL-2. III. Early reductions in donor T cell subsets and expansion of a $CD3^+CD4^-CD8^-$ cell population. *J Immunol* 1992; **148**: 3746.
33. Umland SP, Razac S, Nahrebne DK, Seymour BW. Effects of *in vivo* administration of interferon (IFN)-$\gamma$, anti-IFN-$\gamma$, or anti-interleukin-4 monoclonal antibodies in chronic autoimmune graft-versus-host disease. *Clin Immunol Immunopathol* 1992; **63**: 66.
34. Wit D de, Mechelen M van, Zanin C, *et al.* Preferential activation of Th2 cells in chronic graft-versus-host reaction. *J Immunol* 1993; **150**: 361.
35. Abbas AK, Lichtman AH, Pober JS, eds. *Cellular and Molecular Immunology.* 1991; 231.
36. Antin JH, Ferrara JL. Cytokine dysregulation and acute-graft-versus-host disease. *Blood* 1992; **80**: 2964.
37. Finkelman FD, Katona IM, Mosmann TR, Coffman RL. IFN-$\gamma$ regulates the isotypes of Ig secreted during *in vivo* humoral immune responses. *J Immunol* 1988; **140**: 1022.
38. Bolling SF, Kunkel SL, Lin H. Prolongation of cardiac allograft survival in rats by anti-TNF and cyclosporine combination therapy. *Transplantation* 1992; **53**: 283.
39. Hervé P, Flesch M, Tiberghien P, *et al.* Phase I–II trial of a monoclonal anti-tumor necrosis factor alpha antibody for the treatment of refractory severe acute graft-versus-host disease. *Blood* 1992; **79**: 3362.
40. Hock H, Dorsch M, Diamantstein T, Blankenstein T. Interleukin-7 induces $CD4^+$ T cell-dependent tumor rejection. *J Exp Med* 1991; **174**: 1291.
41. Tilg H, Ceska M, Vogel W, Herold M, Margreiter R, Huber C. Interleukin-8 serum concentrations after liver transplantation. *Transplantation* 1992; **53**: 800.
42. Hervé P, Wijdenes J, Bergerat JP, *et al.* Treatment of corticosteroid resistant acute graft-versus-host disease by *in vivo* administration of anti-interleukin-2 receptor monoclonal antibody (B-B10). *Blood* 1990; **75**: 1017.

ACKNOWLEDGEMENTS. The authors thank Drs A. C. T. M. Vossen and W. A. Noort for valuable discussions, Dr R. Debets for help in the B9 assay, Mr T. M. van Os for graphic design, Mr J. Brandenburg for animal care and Ms J. M. J. Quartel for typing the manuscript. This study was supported by a grant from the Interuniversity Institute for Radiation Pathology and Radiation Protection (IRS), Leiden, The Netherlands.

**Received 21 October 1993; accepted in revised form 17 November 1993**
